# Gastropods and Insects Prefer Different *Solanum dulcamara* Chemotypes

**DOI:** 10.1007/s10886-018-0979-4

**Published:** 2018-07-02

**Authors:** Onno W. Calf, Heidrun Huber, Janny L. Peters, Alexander Weinhold, Yvonne Poeschl, Nicole M. van Dam

**Affiliations:** 1 Molecular Interaction Ecology, Institute for Water and Wetland Research (IWWR), Radboud University, Heyendaalseweg 135, 6525 AJ Nijmegen, the Netherlands; 2 Experimental Plant Ecology, Institute for Water and Wetland Research (IWWR), Radboud University, Heyendaalseweg 135, 6525 AJ Nijmegen, the Netherlands; 3 Molecular Plant Physiology, Institute for Water and Wetland Research (IWWR), Radboud University, Heyendaalseweg 135, 6525 AJ Nijmegen, the Netherlands; 4 German Centre for Integrative Biodiversity Research (iDiv) Halle-Jena-Leipzig, Deutscher Platz 5e, 04103 Leipzig, Germany; 5 Institute of Computer Science, Martin Luther University Halle-Wittenberg, Von-Seckendorff-Platz 1, 06120 Halle, Germany; 6 Institute of Biodiversity, Friedrich Schiller University Jena, Dornburger-Str. 159, 07743 Jena, Germany

**Keywords:** Plant-herbivore interaction, HPLC-qToF-MS, Induced responses, Steroidal glycosides, Eco-metabolomics

## Abstract

*Solanum dulcamara* (Bittersweet nightshade) shows significant intraspecific variation in glycoalkaloid (GA) composition and concentration. We previously showed that constitutive differences in overall GA levels are correlated with feeding preference of the grey field slug (GFS; *Deroceras reticulatum*). One particularly preferred accession, ZD11, contained low GA levels, but high levels of previously unknown structurally related uronic acid conjugated compounds (UACs). Here we test whether different slug species as well as insect herbivores show similar feeding preferences among six *S. dulcamara* accessions with different GA chemotypes. In addition, we investigate whether slug feeding can lead to induced changes in the chemical composition and affect later arriving herbivores. A leaf disc assay using greenhouse-grown plants showed that three slug species similarly preferred accessions with low GA levels. Untargeted metabolomic analyses showed that previous slug feeding consistently increased the levels of N-caffeoyl-putrescine and a structurally related metabolite, but not the levels of GAs and UACs. Slug-induced responses only affected slug preference in one accession. A common garden experiment using the same six accessions revealed that ZD11 received the highest natural gastropod feeding damage, but suffered the lowest damage by specialist flea beetles. The latter preferred to feed on accessions with high GA levels. Our study indicates that different selection pressures imposed by generalist gastropods and specialist insects may explain part of the observed chemical diversity in *S. dulcamara*.

**Electronic supplementary material:**

The online version of this article (10.1007/s10886-018-0979-4) contains supplementary material, which is available to authorized users.

## Introduction

Plants display a significant degree of chemical diversity. Not only among plant species, but also within a single species a multitude of natural chemotypes may exist. A well-studied example for within species chemical diversity are the glucosinolates in *Arabidopsis thaliana*. A study on 39 ecotypes of *A. thaliana* showed that each ecotype is characterized by a specific glucosinolate profile which is a subset of 34 different glucosinolates (Kliebenstein et al. 2001). Another example comes from *Jacobaea vulgaris* (Syn. *Senecio jacobaea*). Plants collected in 11 European populations show a high diversity in their pyrrolizidine alkaloid composition (Macel et al. 2004). It was postulated that the vast intraspecific chemical variation observed in plants today, to a great extent has resulted from co-evolutionary processes with the many members of the herbivore community (Hartmann 1996). Whether a particular chemical compound indeed acts as a defence depends on the level of specialization and feeding strategy of the herbivore species present (Ali and Agrawal 2012). Herbivore communities are not homogenous in time and space. Intraspecific chemical variation may thus result from local or temporal variation in herbivore community composition (Jones and Firn 1991; Speed et al. 2015).

Many chemotypes were found to be heritable (see for example: Macel et al. 2004; van Dam and Baldwin 2003; van Leur et al. 2006). In addition to these genetically fixed differences in constitutive defences, plants are able to induce defences upon herbivory (Howe and Jander 2008; Walling 2000). This enables plants to tailor their response to specific herbivores. Moreover, it allows them to optimize resource allocation to growth or defence under conditions characterized by variable herbivore pressure (Vos et al. 2013; Wittstock and Gershenzon 2002). Herbivore-induced responses can have far-reaching consequences for other members of the herbivore community (Kessler and Halitschke 2007; Poelman et al. 2010). So far, induced responses have mainly been studied in relation to insect herbivory, whereas the effects of gastropod feeding damage are much less well understood. The little information there is suggests that slug feeding, and even their mucus, induces similar responses as chewing insect herbivores and pathogens do (Falk et al. 2014; Meldau et al. 2014; Orrock 2013). On the other hand, slug-induced volatile emissions by *Brassica rapa* were sufficiently different from those induced by insects to be discriminated by parasitoids hunting for caterpillars (Danner et al. 2017; Desurmont et al. 2016). Taken together, this calls for an experimental assessment whether and how slug feeding changes chemical defence levels.

The Bittersweet nightshade (*Solanum dulcamara*) shows large intraspecific variation in constitutive profiles of steroidal glycoalkaloids (GAs, Calf et al. 2018; Eich 2008; Mathé 1970). The fact that clones of the same plant consistently express the same chemotype suggests that there is a strong genetic background for GA composition (Willuhn 1966). We recently showed that resistance to the grey field slug (GFS, *Deroceras reticulatum*) in *S. dulcamara* is associated with high levels of GAs. One strongly preferred accession, ZD11, was found to have particularly low GA levels. Instead, it contained high levels of previously unknown structurally related steroidal compounds which were conjugated with uronic acid (Calf et al. 2018), rather than the mono- or polysaccharides commonly found as glucoside moieties for GAs (Eich 2008; Milner et al. 2011). The observed association between the uronic acid conjugated compounds (UACs) and slug preference illustrates that not only the GA concentration, but also small changes in the glycoside moiety may have large ecological consequences. This is supported by research on potato (*Solanum tuberosum*), which showed that the biological activity of GAs towards gastropods and insects was particularly associated to differences in the glycosylation of the alkaloid backbone (Smith et al. 2001; Tai et al. 2014).

The question arises how these very susceptible UACs-containing accessions survive in natural populations. Slug feeding can exert a strong selection pressure, especially by affecting seedling survival and seedling establishment (Hanley et al. 1995; Rathcke 1985; Strauss et al. 2009). Given the overall high resistance to GFS in the other *S. dulcamara* accessions investigated, it is surprising that UAC-rich chemotypes are not ‘weeded out’ before setting seed (Calf et al. 2018). Possibly UACs provide resistance to other herbivores than GFS. Both slugs and specialist flea beetles are common in natural *S. dulcamara* populations and can affect plant performance (Calf and van Dam 2012; Lortzing et al. 2016; Viswanathan et al. 2005). Generally it has been found that generalist and specialist herbivores differ in their response to chemical defences (Ali and Agrawal 2012). Whereas generalists avoid plants with high levels of chemical defences, specialists may use the same compounds to recognize their preferred hosts (Miles et al. 2005; van Loon et al. 2002). Thus co-occurring generalists and specialists may both play an important role in the maintenance of chemical diversity by differentially selecting high and low GA *S. dulcamara* accessions and affecting their performance. Like many other plant species, *S. dulcamara* is known to increase its levels of defences upon insect herbivore feeding (Nguyen et al. 2016; Viswanathan et al. 2005). Whether slugs can also induce defence responses in *S. dulcamara* is as yet unknown. Our previous studies were carried out using leaf discs taken from undamaged plants, which ignores the role of inducible defence responses. Possibly, the constitutively susceptible accessions invest more in induced responses, thereby saving resources for growth in absence of herbivores (Strauss et al. 2002; Vos et al. 2013). Whether or not this applies, should be tested by assessing the preference for different chemotypes after exposure to slug feeding.

Here we report on a series of experiments designed to test whether *S. dulcamara* accessions, representing different GA chemotypes, are uniformly resistant to different herbivores under greenhouse and field conditions. First, we tested whether constitutive variation among chemotypes similarly affects feeding preferences of three different slug species. Moreover, we tested whether slug herbivory affects subsequent feeding preference of gastropod and insect herbivores. To do so, we first performed a greenhouse-based preference assay using six *S. dulcamara* accessions. The accessions were chosen to represent the full range of GFS resistant to GFS susceptible chemotypes (Calf et al. 2018). Leaf discs of undamaged plants and plants previously exposed to GFS feeding were offered to three slug species to analyse their preference. The leaf metabolomes of GFS damaged leaves were compared to undamaged leaves to analyse differences in constitutive and induced chemical profiles. Additionally, we set up a common garden experiment in which plants of the same accessions were exposed to the natural herbivore community. Half of the plants were repeatedly treated by *Arion* sp. feeding early in the season. This allowed us to test the preference of different members of the natural herbivore community for slug-sensitive and resistant chemotypes, as well as the effect of early season slug feeding.

## Methods and Materials

### Plant Material

*Solanum dulcamara* is a perennial woody vine with its native range stretching from North-Western Europe, Northern Africa to the Asian region north of the Himalayas. It is considered an invasive weed in Northern America, where it serves as a host for economically important pest insects, such as the Colorado potato beetle (*Leptinotarsa decemlineata*) and potato psyllid *(Bactericera cockerelli)* (Castillo Carrillo et al. 2016; Hare 1983). Slug damage commonly occurs on *S. dulcamara* and thus may constitute a significant selection pressure on chemical defences (Lortzing et al. 2016). Six *S. dulcamara* accessions differing in susceptibility to slug herbivory were selected from a previously performed screening of 95 accessions from the Netherlands (Calf et al. 2018). The accessions used in the present study ranged in resistance to the grey field slug from low to highly preferred in the following sequence: GD04-VW08-OW09-TW01-FW09-ZD11 (Calf et al. 2018). Clones of these six accessions were obtained by means of stem cuttings which were potted into individual pots (11 × 11 × 12 cm, L x W x H) containing potting soil (Lentse Potgrond nr. 4, Horticoop, Katwijk, the Netherlands) supplemented with 4 g L^−1^ slow-release fertilizer (Osmocote® Exact Standard, Everris International B.V., Geldermalsen, the Netherlands). Plants were grown in a greenhouse in net cages to prevent insect infection (Rovero 0.3 mm gauze, 7.50 × 3 × 2.75 m, L x W x H) at a 16-h photoperiod and minimum temperatures set to 20 °C/17 °C (day/night). Light levels were supplemented with 1000 W Sodium lamps (Philips GreenPower, Amsterdam, the Netherlands) fixed above the cages providing ~280 μmol m^−2^ s^−1^ light intensity at the plant level.

### Slugs

Three different slug species were used for preference assays, namely the grey field slug (GFS; *Deroceras reticulatum*) and two species complexes belonging to the family Arionidae. Because slugs in the Arionidae family can only be accurately identified by their genitalia, we broadly classified them as *Arion rufus/ater* (ARA) and *Arion fuscus/subfuscus* (AFS). Being relatively small, adults measure about 4 cm, GFS is well suited to be used in small scale preference assays. ARA and AFS grow much larger, adults measure up to 15 cm, which is why we used juveniles of these species. All three slug species are believed to be generalist herbivores, which most commonly feed on fresh leaves, fruits and seedlings. However, this does not imply that they show the exact same preferences.

Slugs were frequently collected in fields and gardens in the vicinity of Nijmegen (the Netherlands) and individually kept in clear 50 ml plastic containers (6 cm Ø, www.der-verpackungs-profi.de GmbH, Göttingen, Germany) lined with sieved (2 mm mesh) humid potting-soil. Containers were placed in a climate cabinet (Snijders Scientific, Tilburg, the Netherlands) under 16-h photoperiod of ~50 μmol m^−2^ s^−1^ light intensity at temperatures set to 17 °C/14 °C (day/night). Diet consisted of self-grown organic lettuce (Bio Pluksla ‘Mesclun’, Dille & Kamille, Zoetermeer, the Netherlands), which was refreshed twice a week. Containers were cleaned every week by removing faeces, diet residues and excess water. Slugs were transferred to clean containers with fresh soil monthly.

### Experiment 1: Greenhouse Experiment

#### Plant Growth and Feeding-Induction Procedure

Plants were grown from stem cuttings (Calf et al. 2018), replicates of one accession are hereafter called clones. All plants were 38 days old and at least 60 cm tall when used for experiments. Plants were fitted with clip cages, either containing one adult GFS (feeding-induction treatment, 4 clones/accession) or empty clip cages (control treatment, 4 clones/accession) on the tip of two leaves (number 10 and 11 from the apex) to prevent total leaf consumption. The cages and the slugs were removed after 72 h. All plants in the slug treatment showed substantial feeding damage (estimated by eye, > 1 cm^2^). The plants were left for an additional 24 hr before sampling. In preliminary experiments this procedure was found suitable for detecting induced plant responses (OW Calf, unpublished results). The part of the leaf that was outside of the clip cage was used to produce six leaf discs using a cork-borer (1.5 cm Ø), thus creating a total of 48 leaf discs (2 leaves * 4 clones * 6 discs) per treatment group. Leaf veins were avoided while punching out the discs. Leaf discs were pooled per accession and treatment group. The remaining leaf tissue was sampled in liquid nitrogen and stored at −80 °C until further processing for chemical analyses.

#### Preference Assays

One leaf disc of each treatment and accession was placed in a Petri dish (9 cm Ø) with the adaxial (i.e. upper) side down. The Petri dishes were gently sprayed with de-ionised water before, during and after placing the leaf discs to maintain leaf disc turgor. This procedure also ensured that the discs adhered to the Petri dish and stayed at the same position while the slugs were feeding. Each dish contained one disc of each of the six selected accessions in both the constitutive and GFS feeding-induced state, adding up to twelve discs per dish. Remaining leaf discs were discarded. Within each Petri dish, the disc positions were randomised. The positions were printed on a piece of paper which was placed under the transparent dish. Depending on the size of the slugs, either two or three individuals of either GFS, ARA or AFS (*n* = 12 Petri dishes per species) were placed on the lid of each Petri dish. Thereafter the dishes were closed and placed in a climate cabinet under the same conditions as above. After 24 hr, the slugs were removed and the leaf material remaining in the Petri dish was photographed with a 14 cm ruler as scale reference. The consumed area (in mm^2^) was assessed using ImageJ v. 1.48 (Schneider et al. 2012).

#### Metabolic Profiling

An untargeted metabolomics approach was used to assess the effect of GFS feeding-induction on the metabolic profiles of the six *S. dulcamara* accessions. Leaf samples (*n* = 4 per accession and treatment group) were extracted following a procedure derived from (de Vos et al. 2012). In short, fresh leaf material was ground in liquid nitrogen. About 100 mg of each sample was double extracted with respectively 1.0 and 0.9 ml MeOH:Acetate (50/50, v/v %) buffer (pH 4.8) in 2 ml reaction tubes holding two glass beads (5 mm Ø) by shaking in a TissueLyser (Qiagen, Venlo, the Netherlands) at 50 Hz for 5 min followed by centrifugation at 14.000 rpm at 4 °C. Clear supernatants were combined and stored at −20 °C until further processing.

Two sets of diluted crude extracts (1:5 and 1:50) were analysed with an UltiMate™ 3000 Standard Ultra-High-Pressure Liquid Chromatography system (UHPLC, Thermo Scientific) equipped with an Acclaim® Rapid Separation Liquid Chromatography (RSLC) 120 column (150 × 2.1 mm, particle size 2.2 μm, ThermoFisher Scientific) using the following gradient at a flow rate of 0.4 ml/min: 0–2 min isocratic 95% A (water/ formic acid 99.95/0.05 (v/v %)), 5% B (acetonitrile/formic acid 99.95/0.05 (v/v %)); 2–15 min, linear from 5 to 40% B; 15–20 min, linear from 40 to 95% B; 20–22 min, isocratic 95% B; 22–25 min, linear from 95 to 5% B; 25–30 min, isocratic 5% B. Compounds were detected with a maXis impact –quadrupole time-of-flight mass spectrometer (qToF-MS, Bruker Daltonics) applying the following conditions in positive mode: scan range 50–1400 *m*/*z*; acquisition rate 3 Hz; end plate offset 500 V; capillary voltage 3500 V; nebulizer pressure 2 bar, dry gas 10 L min^−1^, dry temperature 220 °C. Mass calibration was performed using sodium formate clusters (10 mM solution of NaOH in 50/50 (v/v %) isopropanol water containing 0.2% formic acid.

Mass spectra of the dataset obtained from analysing the 1:5 diluted samples were processed using XCMS and CAMERA packages in R (Kuhl et al. 2012; Smith et al. 2006). Peak picking and alignment was done within a retention time between 45–700 sec, signal:noise ratio ≥ 50, maximum deviation of 5 ppm, 3 sec retention time window, mass/charge window of 0.005 and minimum occurrence in three out of four samples in at least one treatment group. Resulting features were grouped to belong to the same compound after symmetric retention time correction, within a retention time window of maximum 5 sec and minimum mutual correlation of 0.8. Feature intensities were multiplied by their dilution factor (5) and samples normalised by the fresh weight of the leaf material used for extraction. Missing values were replaced by the treatment group mean value only when one of four replicates was missing or else by a random value between 1 and 7722 (half of the minimum value in the complete dataset). Feature groups, potentially representing single compounds, were reduced to one feature to represent the respective compound in later analysis. This was done by applying the in-house maximum heuristic approach, which selects the feature with the highest intensity in the majority of the samples.

Intensities of the 50 most prominent peaks (signal:noise > 10) were manually identified from the datasets obtained from the 1:5 and 1:50 diluted samples, following the same procedure as described in Calf et al. (2018). Feature intensity values were multiplied by their dilution factor (5 or 50) and values in the 1:5 dilution dataset exceeding the saturation intensity threshold value of 6 × 10^6^ g^−1^ FW were replaced with the corresponding value from the 1:50 dilution dataset. The resulting dataset included all GAs and UACs of interest, which were thus reliably quantified and no subject of random errors caused by automatic data processing. The dataset of abundant features and that processed by XCMS and CAMERA were then merged to a single dataset. Features which were quantified by both procedures, were replaced by the manually retrieved feature intensities. This approach resulted in a dataset with 286 features, hereafter referred to as compounds.

Tandem mass spectrometry (MS^2^) spectra were acquired by injection of samples that contained the highest amount of the compounds of interest (see section below: Statistical Analyses Experiment 1). The separation was achieved by using the same chromatographic conditions as described above. MS^2^ spectra were collected by using the automated MSMS function of the Bruker oToF Control software. Spectra were evaluated for compounds of interest with particular emphasis on fragmentation of the parental compound. Putative identifications were made based on comparison of mass spectra reported in the literature.

#### Statistical Analyses Experiment 1

Absolute leaf disc consumption (mm^2^) in the preference assays of experiment 1 were transformed to preference ranks ranging from 1 (least preferred) to 12 (most preferred) within each Petri dish. Leaf discs with equal damage were assigned the mean of the two consecutive ranks that would have been assigned. Note, when for instance five plants received no damage each would receive rank number 3 using this approach. Data were analysed using nonparametric statistical methods from the R “stats” package (R Core Team 2016). Friedman’s rank sum test was applied to evaluate overall preference for each slug species using the Petri dish number as grouping factor. Chi^2^ tests were performed on the mean ranks for each treatment group to test similarity in preference among different slug species. The effect of the feeding induction treatment was evaluated individually for each *S. dulcamara* accession using Paired Wilcoxon signed rank tests with continuity correction, excluding ties (‘no difference’).

The complete metabolic dataset of 286 compounds was further analysed using the online R-based tool MetaboAnalyst 3.0 (Xia et al. 2015). Principal component analyses (PCA) was performed using log_10_-transformed and auto-scaled intensity values to evaluate the overall metabolic variation among accessions and treatments.

The effect of GFS feeding-induction on metabolic profiles was assessed with discriminant analyses using orthogonal projection models to latent structures (OPLS-DA, Wiklund et al. 2008; Worley and Powers 2013). These models separate metabolic variation correlated with the treatment from random variation among samples, which allowed for reliable selection of compounds of interest which responded to the GFS feeding-induction treatment. First, the intensity value of each compound was normalised to the mean value of the same compound in the control samples of the respective accession. OPLS-DA models were built to compare the log_10_-transformed relative intensity values of all compounds in control samples and GFS feeding-induced samples. Two approaches were used: 1) an overall model was built including all six accessions to assess the shared response (*n* = 24 for each treatment). 2) Individual models for each accession were built to assess accession specific responses (*n* = 4 for each treatment). Predictive significance of the model was assessed with 1000 permutations using cross-validated predictive ability (Q^2^) as performance measure (Westerhuis et al. 2008; Worley and Powers 2013). Compounds of interest were selected from the S-plot with the OPLS-DA models. The S-plot depicts the response (covariance) and reliability of the response (correlation) of each compound in relation to the first predictive component (p) of the model. Thresholds for selection as compound of interest were set to absolute covariance_(p)_ ≥ 1.5 and correlation_(p)_ ≥ 0.5. Venn diagrams were built using the online tool Venny 2.1.0 (Oliveros 2007–2015) and used to illustrate unique and shared metabolic responses among accessions.

Significance of the treatment effect within each accession on the abundance of compounds was further evaluated using Student’s t-tests with application of *P*-value correction for the false discovery rate (FDR) using the Bonferroni method. The list of compounds included the set of pre-selected compounds (four GAs and six UACs), which was supplemented with compounds of interest selected from the S-plot of the overall OPLS-DA model. The abundance of different compounds can only be compared on a relative scale. Therefore, the relative levels of each compound of interest to the maximum mean value across all treatment groups was used for presentation of compound abundance in a heat map (%).

### Experiment 2: Common Garden Experiment

#### Natural Herbivory in a Common Garden

Plants were grown in the greenhouse for 29 days. Plants of similar size and appearance within accessions were planted (*n* = 10 per accession) following a randomised block design in a common garden field plot in the experimental garden of Radboud University in Nijmegen on 14 May 2016. *S. dulcamara* grows as a climbing vine. Therefore, each plant was supported by a cylindrical wire structure (1 mm stainless steel wire, mesh = 20 cm, height = 100 cm, Ø = 50 cm) eight weeks after planting. Because GFS slugs were not available in sufficient numbers at the time, juvenile ARA slugs were used for the feeding-induction treatment on five clones per accession. Slugs were placed in a clip cage on the 10th leaf counted from the apex for 24 hr. This treatment was repeated twice a week for three consecutive weeks, whereby the treated leaf was chosen following the same criteria. When the designated leaf was not/no longer available due to excessive herbivore feeding, the leaf on the internode over the designated leaf was used.

All species that were observed on the plant throughout the field season of 2016 were recorded. The plants were monitored from late-May to October 2016 (calendar weeks 21–41), with the exception of weeks 25, 36 and 37. The numbers of all invertebrate organisms on each plant in the plot were recorded twice every week by surveying the entire plant for one minute. The small size and cryptic life strategy of most observed herbivore species makes it unlikely to record them all at each census date. Moreover, daily variation in weather conditions affects their activity level and thus the likelihood of observing them. To compensate for this day-to-day variation, only the maximum number of each species on each plant recorded each week were entered into the data analyses (Table 1). The percentage gastropod and flea beetle damage on each plant was scored once every week. The damage was classified in six equally sized damage classes, ranging from no damage at all (class 0) to severe damage (class 5). In the latter category >75% of the leaf material had been removed which often had led to leaf and stem deformations (Table S1). Gastropod feeding causes large holes often starting from the edge of the leaf. These could be identified as slug damage by the presence of typical bite marks of the radula and slime residues. Slug damage was estimated over the entire plant, thereby excluding the leaves that had been subject to the ARA treatment. Flea beetle feeding also causes a specific damage pattern, seen as numerous small holes, which are also described as a shot-gun pattern. Fresh flea beetle damage was only assessed on the top 20 cm of all shoots, where feeding typically occurs.

**Table 1:** Results of OPLS-DA models comparing the overall or individual metabolic profiles of six *Solanum dulcamara* accessions that were either left undamaged or induced by feeding damage of the grey field slug (*Deroceras reticulatum*). The number of plants per treatment (n), cross-validated predictive ability (Q^2^ in %), explained variance by the first predictive component (R^2^X_(p)_ in %), explained variance by the first orthogonal component (R^2^X_(o)_ in %) and number (n) of up- and down-regulated compounds of interest are provided for each model

Model	n	Q^2^	R^2^X_(p)_	R^2^X_(o)_	n Up	n Down
Overall	24	71.6^***^	3.9	8.9	3	0
GD04	4	43.0	17.1	17.8	19	29
VW08	4	67.8^+^	20.4	14.3	42	32
OW09	4	65.4^+^	22.1	18.6	41	28
TW01	4	75.4^*^	27.3	19.0	33	36
FW09	4	70.3^*^	22.1	15.0	42	18
ZD11	4	74.0^*^	23.2	19.5	31	40

Symbols indicate significance of the cross-validated model based on 1000 permutations (***P < 0.001, * *P* < 0.0*5,*^*+*^*P* < 0.1)

Plant size and herbivore abundance have been shown to be positively correlated (Castells et al. 2017; Wei et al. 2015; Windig 1993). Therefore we recorded the total plant size, calculated as the summed length of all living shoots, nine times throughout the growing season. This allowed us take variation in plant size into account when comparing herbivore abundance. Plants that were completely consumed in the course of the experiment (three ZD11 individuals), were excluded from further analyses.

#### Statistical Analyses Experiment 2

Similar to the greenhouse assays described above, the observed damage classes of gastropod and flea beetle damage in the common garden observations were transformed to preference ranks ranging from 1 (least preferred) to 60 (most preferred) within the garden plot. Data were analysed using nonparametric statistical methods from the R “stats” package (R Core Team 2016). Unpaired Mann Whitney and Kruskal-Wallis rank sum tests were performed to test for the effect of slug feeding-induction and the differences between accessions for each week, respectively. Two-way ANOVA with Tukey’s post-hoc test was performed to test for differences in final total stem length between accessions. Total stem lengths were square-root transformed to ensure normal distribution and homogeneity of the variances.

## Results

### Experiment 1: Greenhouse Experiment

#### Slug Preference Assays

**Fig. 1:**
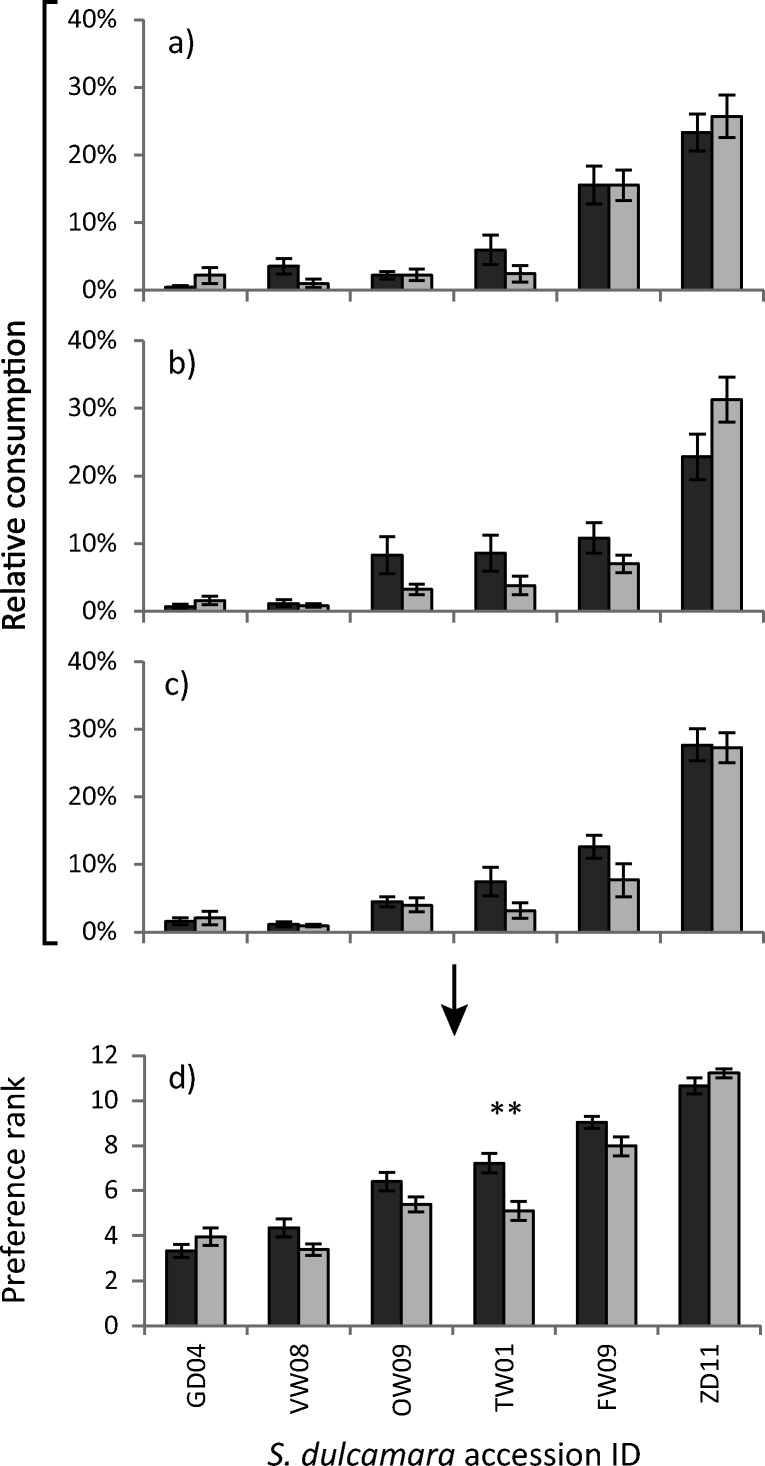
Mean relative consumption (±SE) by (**a**) *Deroceras reticulatum* (*n* = 12)*;* (**b**) *Arion rufus/ater* (*n* = 12) and (**c**) *Arion fuscus/subfuscus* (*n* = 12) as well as (**d**) the combined mean preference rank (±SE) of all three slug species (*n* = 36) on leaf discs of *Solanum dulcamara* accessions that were either left undamaged (control; dark grey bars) or induced by feeding damage of *D. reticulatum* (72 hr of feeding followed by 24 hr relaxation, light grey bars). Asterisks indicate a significant treatment effect according to Wilcoxon Signed Ranks test with Bonferroni correction for multiple comparisons (** *P* < 0.01)

The three different slug species showed the same preference for the six *S. dulcamara* accessions. Their preference was independent of whether the discs were from plants previously subjected to GFS feeding-induction (Fig. 1a–c, GFS vs ARA: χ^2^ = 2.325, df = 11, *P* = 0.997, GFS vs AFS: χ^2^ = 5.689, df = 11, *P* = 0.893, ARA vs AFS: χ^2^ = 2.535, df = 11, *P* = 0.996). By pooling the preference ranks of the three different species, we found that slugs overall showed a significant preference among accessions (Fig. 1d, Friedman χ^2^ = 240.3, df = 11, *P* < 0.001). Accessions were preferred in the exact same order as reported in the original study from which they were selected (Calf et al. 2018), with ZD11 being the most preferred accession. Previous GFS feeding significantly reduced subsequent slug feeding preference only for accession TW01 (Fig. 1d).

#### Metabolomic Profiling of GFS Feeding-Induced Responses

**Fig. 2:**
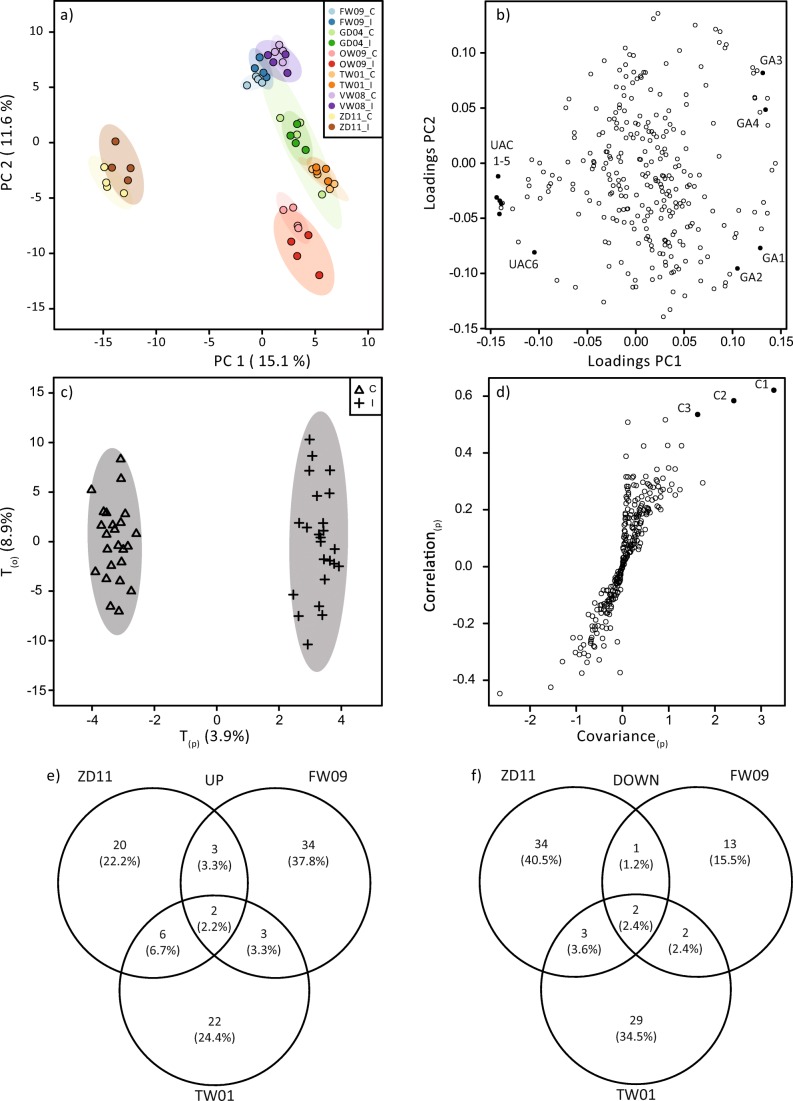
Metabolic profiling of leaves taken from six *Solanum dulcamara* accessions that were either left undamaged (C) or induced by feeding damage of *Deroceras reticulatum* slugs (I; 72 hr of feeding followed by 24 hr relaxation). Profiles are based on 286 compounds retrieved by untargeted HPLC-qToF-MS analyses. **a** PCA scores plot of accessions subjected to both treatments (*n* = 4 clones/accession/ treatment); (**b**) compound loadings in the PCA scores plot. Filled dots indicate pre-selected compounds of interest (GA1–4 and UAC1–6); (**c**) T-score plot of the first predictive component (p) and orthogonal component (o) in the overall OPLS-DA model including all six accessions (*n* = 24/treatment); (**d**) S-plot for compound importance in the same OPLS-DA model. Filled dots indicate selected compounds of interest (C1-C3); panel (**e)** and (**f**) Venn-diagrams of respectively up- and downregulated compounds selected from three significantly cross-validated individual accession OPLS-DA models

Accessions differed with respect to their metabolic profiles. A PCA on the metabolomes of the six *S. dulcamara* accessions revealed that accession ZD11 clustered separately from the other five accessions. ZD11 mainly separated on the first principal component (PC1), which explained 15.1% of the overall variation (Fig. 2a). The other five accessions separated on the second principal component (PC2, 11.6%), with exception of VW08 and FW09. Separation in the PCA was mainly based on the abundance of UACs (PC1) and tomatidenol/solasodine-type GAs (PC2, Fig. 2b). This is in accordance with chemotypic data from the original study based on which the accessions were selected (Calf et al. 2018). Samples of plants subjected to GFS feeding always clustered with their respective undamaged controls.

Because slug-induced responses may be too subtle to be visible in the overall PCA model, OPLS-DA models were built to compare the metabolomes of control and GFS feeding-induced plants from the whole data set, as well as for each accession separately (Table 1). The overall model, including all six accessions, revealed that the metabolomes of damaged and undamaged plants were significantly different (Fig. 2c and Table 1). The model had significant (*P* < 0.001) cross-validated predictive ability of 71.6%. However, the predictive component (p) explained merely 3.9% of the total variance, whereas 8.9% of total variance was explained by the orthogonal component (o). This illustrates that only a small portion of the metabolome showed a shared response to the treatment and more variation is explained by differences among accessions. Three compounds of interest (C1-C3) were selected that showed the most consistent and reliable response among accessions to the GFS feeding-induction treatment, based on the S-plot with the OPLS-DA model (Fig. 2d and Table S2). C1 and C2 were putatively identified as two isomers of the phenolamide N-caffeoyl-putrescine (Table S3). C3 is a related compound which was proposed to be an N-caffeoyl-putrescine metabolite in a study by Li et al. (2015).

Induced responses in each accession were tested using individual models comparing undamaged control and slug-induced plants per accession. Interestingly, only the models of the three most sensitive accessions, ZD11, FW09 and TW01 showed significant predictive ability after cross-validation (*P* < 0.05; Q^2^ = 70.3–75.4%; Table 1). The three significant models explained between 22.1–27.3% of the total variance, which is much higher than the overall model. This illustrates that responses to GFS feeding induction are relatively specific for each accession. Compounds of interest of the individual models were selected based on the same criteria as the overall model. The majority of regulated compounds in accession FW09 were upregulated, whereas a similar or higher number of compounds was downregulated in ZD11 and TW01. However, there was very little overlap in the compounds that were up- (Fig. 2e) or downregulated (Fig. 2f) among these three accessions, which again points to accession specific responses.

**Fig. 3:**
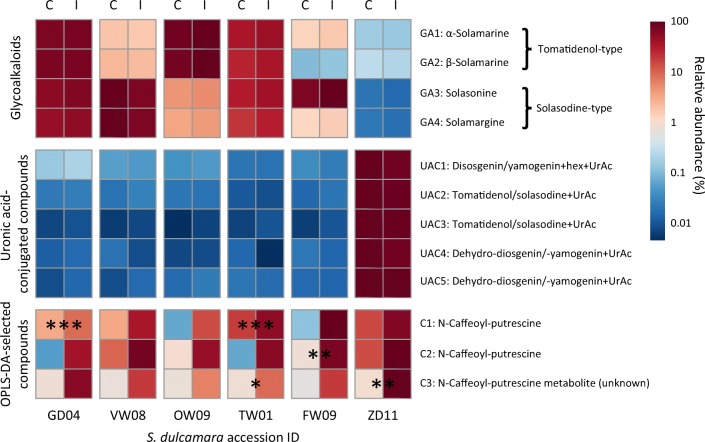
Mean log_10_-transformed peak intensities relative to the maximum observed intensity of selected compounds of interest in leaves of six *Solanum dulcamara* accessions that were either left undamaged (C, *n* = 4 clones / accession) or induced by feeding damage of *Deroceras reticulatum* slugs *(*I; 72 hr of feeding followed by 24 hr relaxation, *n* = 4 clones/accession). The peak intensities of four steroidal glycoalkaloids, five uronic acid conjugated compounds and three selected regulated compounds are shown. Asterisks indicate significant treatment effects according to FDR-adjusted *P-*values of independent t-tests for each accession (***P < 0*.*001, ** *P* < 0*.*01, * *P* < 0*.*05)

GFS feeding-induction did not affect the abundance of GAs and UACs in any accession (Fig. 3). However, each accession, except for VW08 and OW09, showed upregulation of at least one of the previously selected phenolamides.

### Experiment 2: Common Garden Experiment

None of the analyses on herbivore numbers or damage revealed significant effects of previous slug feeding (unpaired Wilcoxon signed-rank test, *P* > 0.05, data not shown). For this reason, the data for control and slug-induced plants were pooled for further analyses.

#### Overall Herbivore Abundance

**Fig. 4:**
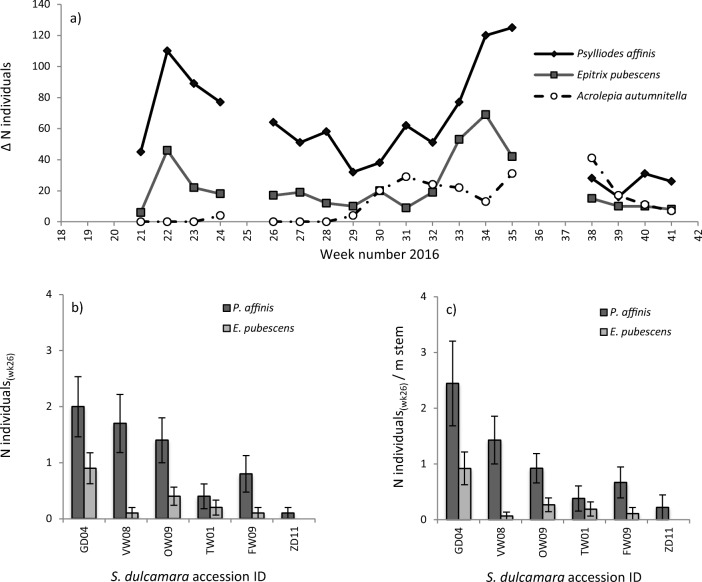
Abundance of specialist leaf herbivores in common garden experimental area. **a** Seasonal pattern of occurrence of three species of specialist leaf herbivores from late-May to October 2016 (calendar week 21–41). Lines are broken at the time points that the plants were not monitored; panel (**b)** and **(c**) Means (± SE) of respectively the absolute numbers and relative numbers to stem length (± SE) of two species of flea beetles in week 26 on six *Solanum dulcamara* accessions

In total we observed 20 types of organisms on *S. dulcamara* in the common garden experiment (Table 2). Eight of the observed insect species, mainly beetles, belonged to specialist herbivores on Solanaceae. Seven were classified as ‘other herbivores’. This group mainly included generalists, as well as *Lilioceris lilii,* which is a specialist on plants in the Liliaceae family, but also a facultative visitor of *S. dulcamara* (Cox 2001). Six other herbivores were classified as third trophic level insects, mainly predators of arthropod herbivores. To analyse differences among accessions, we focussed on the most abundant leaf feeding specialist insects, namely *Psylliodes affinis, Epitrix pubescens* and *Acrolepia autumnitella* (Table 2). For each of these species we counted >30 individuals on at least one of the census dates. The remaining herbivore species either were found too infrequently to be reliably analysed, or they fed on flowers, such as the larvae of *Contarinia solani* gall midges and *Pria dulcamarae* sap beetles*.* The flea beetle *P. affinis* was the most abundant herbivore, with on average > 1 individual per plant each week (Table 2). The second most abundant herbivore was another flea beetle *E. pubescence*, which abundance was about third of that of *P. affinis*. Over the growing season, the abundance of both flea beetle species was synchronised. There were two generations of adults; a spring generation with peak abundance at the end of May (week 22) and a mid-summer generation peaking in the end of August (week 33–35, Fig. 4a). The larvae of the specialist moth *A. autumnitella* are leaf miners. They appeared to have three generations, with peak abundances of new mines in mid-June, early August and September (week 24, week 31 and weeks 35–38, Fig. 4a). Their peak abundance increased with time, which indicates a typical population increase over the season. Slugs and snails were not frequently observed because of their nocturnal feeding habit. Differences in gastropod abundance among accessions could therefore not be tested.

**Table 2:** The type of organisms, their mode of feeding and mean observed number on each of 60 *Solanum dulcamara* plants during each of 19 weeks of common garden observations in 2016. The maximum number observed on all plants at one census date is given in brackets

Common name	Species	Order, Family	Feeding type	Mean n individuals / plant / week
A. Specialist herbivores				
Bittersweet leaf miner moth	*Acrolepia autumnitella*	Lepidoptera, Glyphipterigidae	Leaf mining	0.206 (41)
Nightshade gall midge	*Contarinia solani*	Diptera, Cecidomyiidae	Gall-mining larvae in flowers	0.522 (205)
Nightshade flea beetle	*Epitrix pubescens*	Coleoptera, Chrysomelidae	Leaf chewing	0.375 (69)
Colorado potato beetle	*Leptinotarsa decemlineata*	Coleoptera, Chrysomelidae	Leaf chewing	0.004 (2)
Bittersweet sap beetle	*Pria dulcamarae*	Coleoptera, Nitidulidae	Adults on pollen, larvae in flower anthers	0.133 (26)
Potato flea beetle	*Psylliodes affinis*	Coleoptera, Chrysomelidae	Leaf chewing	1.019 (125)
Bittersweet flea beetle	*Psylliodes dulcamarae*	Coleoptera, Chrysomelidae	Leaf chewing	0.044 (10)
B. Other herbivores				
Scarlet lily beetle	*Lilioceris lilii*	Coleoptera, Chrysomelidae	Leaf chewing	0.006 (3)
Cicadas*		Hemiptera	Cell sucking	0.037 (8)
Heteropteran bugs		Hemiptera	Cell sucking	0.125 (23)
Aphids**		Hemiptera, Aphididae	Sap sucking	NA
Jumping plant lice***		Hemiptera, Psyllidae/Triozidae	Sap sucking	NA
Lepidopteran larvae****		Lepidoptera	Leaf chewing	0.005 (4)
Typical snails		Pulmonata, Helicidae	Leaf chewing	0.023 (6)
C. Third trophic level				
Ladybirds		Coleoptera, Coccinellidae	Predator	0.014 (10)
Hoverflies		Diptera, Syrphidae	Predator	0.006 (3)
Ants		Hymenoptera, Formicidae	Predator	0.010 (5)
Common parasitoids		Hymenoptera, Ichneumonidae	Predator	0.118 (20)
Green lacewings		Neuroptera, Chrysopidae	Predator	0.004 (1)
Red velvet mite		Trombidiformes, Trombidiidae	Predator	0.166 (51)

*Early season significant number of meadow spittlebugs *(Philaenus spumarius)*

**Not recorded in absolute numbers, but instead as being present or absent, only abundant in start of season

***Possibly the potato specialist (Bactericera cockerelli) is among these, only noticed in last weeks of observations

****Fair number supposedly belonging to family of leafroller moths *(Tortricidae)*

#### Accession Specific Herbivore Abundance

The distribution of insects was evaluated in absolute numbers per plant as well as relative to stem length. The latter was done because total stem length was found to differ significantly among accessions at the end of the growing season (One-way ANOVA_Wk41_: df = 5, F = 9.25, *P* < 0.001, Fig. S1). Absolute and relative flea beetle abundance significantly varied among accessions (Table S4). Only in week 26 both the absolute and relative numbers of beetles were differently distributed among accessions (Kruskal Wallis Test *P* < 0.05, Fig. 4b, c, Table S4). In that week, most individuals were observed on accession GD04. However, the accession on which most flea beetles were observed differed throughout the season. The lowest numbers of *P. affinis* and *E. pubescens* were always observed on accession ZD11 (Kruskal Wallis Test, *P* < 0.05, Table S4). In contrast to flea beetle distribution, individuals of the moth *A. autumnitella* were not differently distributed among accessions on any census date (Kruskal Wallis χ^2^ < 0.99, df = 4, *P* > 0.05, data not shown).

#### Gastropod and Flea Beetle Damage Scores

**Fig. 5:**
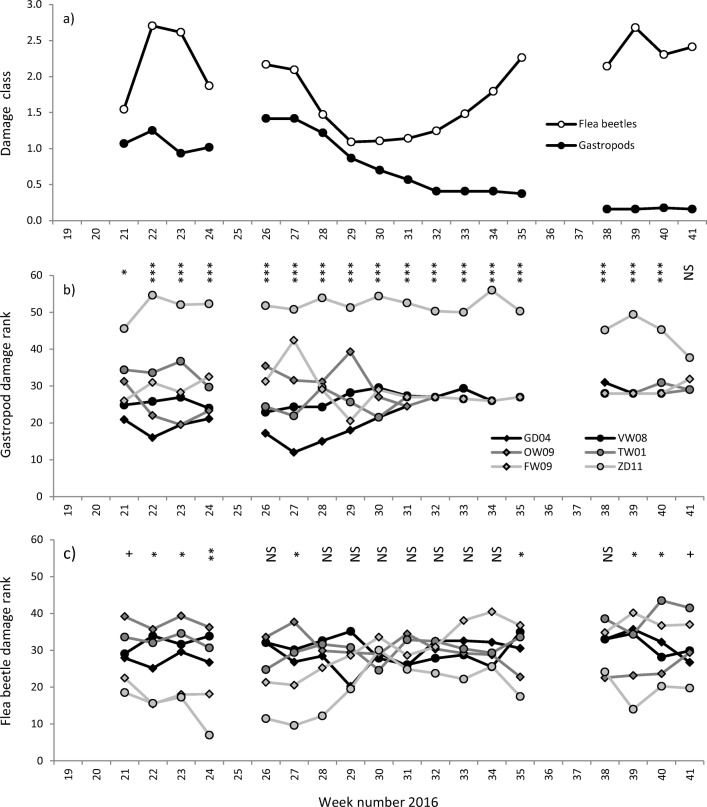
Seasonal pattern of herbivory as observed on six *Solanum dulcamara* accessions (*n* = 10) in a common garden experiment from late-May to October 2016 (calendar week 21–41). **a** overall mean gastropod and flea beetle damage classes on all accessions, (**b**) mean gastropod and **c**) mean flea beetle damage ranks derived from scored damage classes per accession. Lines are broken at the time points that the plants were not monitored. Symbols indicate significant differences according to Kruskal-Wallis Signed Rank test (*** *P* < 0*.*001, ** *P* < 0*.*01, * *P* < 0*.*05, ^+^*P* < 0.10)

We recorded gastropod and flea beetle damage in damage classes, as a measure for herbivore preference (Fig. 5, Table S1). Overall gastropod damage was greatest at the start of the experiment and gradually decreased over the season (Fig. 5a). Flea beetle damage (Fig. 5a) was strongly synchronised with flea beetle abundance (Fig. 4a).

Gastropod damage significantly differed among accessions over the entire season, except for the last census week, and was always the greatest on accession ZD11 (Fig. 5b). Three plants of ZD11 died because of excessive gastropod feeding (1 in week 32, 2 in week 38). These plants were excluded from further analyses. Accession GD04 showed the lowest gastropod damage in the first half of the season. The gastropod damage ranks of GD04, OW09, TW01 and FW09 significantly changed with time (Kruskal-Wallis χ^2^ > 41.80, df = 17, *P* < 0.001), with average rank numbers becoming more equal over time. This effect is explained by the fact that overall gastropod damage decreased over the season (Fig. 5a). Therefore fewer plant incurred any damage and were assigned the same average rank number. Also after exclusion of ZD11 from the statistical analyses, gastropod damage remained different among accessions for the first half of the season (week 22, 23, 26–29, Kruskal-Wallis χ^2^ > 9.70, df = 4, *P* < 0.05, data not shown).

Flea beetle damage differed among accessions only at the start (week 21–24 and 27) and the end (week 35 and 39–41) of the season (Fig. 5c). Generally, accession ZD11 received the lowest amount of flea beetle damage, which is in line with the low flea beetle abundance observed on this accession (Fig. 4b and c, Table S4), but opposite to gastropod feeding preference (Fig. 5b and Fig. S2). The flea beetle damage rank changed throughout the season for FW09 (Kruskal-Wallis χ^2^ > 49.64, df = 17, *P* < 0.001). The mean damage rank of this accession was among the lowest in spring (week 21–24), but gradually increased towards the end of the season.

## Discussion

Our study revealed that generalist gastropods and specialist flea beetles display opposite feeding preferences among six *S. dulcamara* accessions with distinctive GA chemotypes. Previous feeding by slugs had little effect on this preference. The UAC-rich accession ZD11, which was uniformly preferred by slugs in both experiments, suffered the least damage by specialist flea beetles in the field. Gastropods and flea beetles thus seem to exert opposing selection pressures on chemical diversity in GA and UAC profiles.

All three slug species tested in the greenhouse experiment showed highly similar preferences for accessions with low GA levels. These results are similar to those of the original experiment with GFS only, based on which the accessions were selected (Calf et al. 2018). The gastropod feeding damage incurred by the different accessions in the common garden experiment confirmed that gastropods also prefer low GA accessions under field conditions. Our findings are in line with previous studies, reporting effects of several types of alkaloids on gastropod feeding damage (Bog et al. 2017; Smith et al. 2001; Speiser et al. 1992). This suggests that the observed preference of generalist gastropods is driven by differences in GA levels among *S. dulcamara* accessions. Interestingly, the preference and damage patterns of flea beetles were diametrically different. The accession that was the most preferred by the slugs was the least preferred by these specialist insect herbivores, which are commonly associated with *S. dulcamara* (Calf and van Dam 2012; Castillo Carrillo et al. 2016; Viswanathan et al. 2005).

Specialists often use the presence of specific chemical defences, such as GAs, to recognise their host (van Loon et al. 2002). Seen the particular taste of GAs – which gave *S. dulcamara* its common name “bittersweet nightshade” – it is likely that herbivores use them as cues. Possibly, the flea beetle species observed in this study did not feed on UAC-rich accession ZD11, because of the lack of GAs as a feeding stimulant. Moreover, specialists often possess specific mechanisms to deal with their host’s toxins. The Colorado potato beetle, which also commonly occurs on *S. dulcamara* (Calf and van Dam 2012; Hare 1983), was shown to excrete GAs (Armer 2004). Also the larvae of tortoise beetles feeding on *S. dulcamara* in northern America excrete GAs. They even use them for their own benefit by incorporating the compounds in faecal shields as defence against predators (Vencl et al. 1999). Sequestration of plant chemical defences in the insect’s haemolymph frequently occurs in specialist insects, including flea beetles (Beran et al. 2014; Nishida 2002). However, this has not been observed for *Solanum* GAs yet (Opitz and Müller 2009). We did not explicitly test for toxic effects of GAs and UACs on different herbivores, which would require measurements of herbivore performance. This question could be assessed by bio-assays in which herbivores are offered different concentrations of GAs and UACs in artificial diets, or genetically modified *S. dulcamara* plants with altered GA concentrations (Voelckel et al. 2001).

Previous slug damage significantly reduced the relative feeding preference of slugs in only one accession (TW01) in the greenhouse experiment and not at all in the experimental garden. Moreover, none of the accessions showed a significant increase in GA levels within 96 hr after the onset of slug feeding. Induction of the biosynthesis pathway of GAs is regulated by jasmonate-responsive transcription factors (Thagun et al. 2016). Two earlier studies showed that GFS, in contrast to other gastropods, secretes salicylic acid in its locomotion mucus (Kästner et al. 2014; Meldau et al. 2014), which attenuates jasmonic acid dependent defence signalling (Schweiger et al. 2014; Thaler et al. 2012). Besides, the induction of secondary metabolites is dynamic in time and induced responses are known to differ between damaged and undamaged tissues (Chung et al. 2013; Erb et al. 2009; Mathur et al. 2011; van Dam and Raaijmakers 2006). It may thus well be that changes in GA abundance are only observed in other tissues or at a later time point. In the garden experiment, we had to use ARA instead of GFS to induce the plants, which may have elicited different responses. Additionally, the natural gastropod feeding occurring immediately after transplantation to the garden may have overruled the effect of our induction treatment with single slugs. Both factors may have prevented us from finding effects of our controlled slug induction treatment in the common garden experiment. Further research on the dynamics of local and systemic slug-induced resistance, in combination with metabolic profiling, is needed to better understand defence regulation upon slug feeding under greenhouse and field conditions.

Our untargeted metabolomic analyses revealed that only the metabolomes of the three most susceptible accessions (TW01, FW09 and ZD11) were significantly changed in response to slug feeding. This suggests that these accessions may rely more on induced responses, potentially saving resources for growth in absence of herbivores (Strauss et al. 2002; Vos et al. 2013). Alternatively, the slugs may have caused more feeding damage on these susceptible accessions, thereby triggering a stronger response. This may also explain the accession specific responses, as illustrated by the little overlap in the metabolites that were up- or downregulated. Only the levels of two isomers of the phenolamide caffeoyl-putrescine and a related metabolite were consistently increased among *S. dulcamara* accessions. Phenolamides, also known as hydroxycinnamic acid amides, are commonly found in plants. These compounds have a diverse role in plant development, such as flowering and adaptive responses to stress conditions (reviewed by Bassard et al. 2010; Edreva et al. 2007). Phenolamide metabolism is particularly well-studied in wild tobacco, *Nicotiana attenuata*. Various phenolamides in this species, including caffeoyl-putrescines, are induced in local and systemic tissue after insect feeding, as well as in response to UV-B radiation (Demkura et al. 2010; Gaquerel et al. 2014). Because the polyamides were induced in slug-sensitive and -resistant *S. dulcamara* accessions alike, these compounds can serve as a biochemical indicator for defence responses. Based on our experiments we cannot correlate them to (induced) resistance to slugs, as we observed only in one accession that slug damage reduced slug preference. However, their induction may have consequences for other organisms, as several studies correlated high levels of phenolamides with increased resistance to herbivores or pathogens (Fixon-Owoo et al. 2003; Kaur et al. 2010; Tai et al. 2014; Tebayashi et al. 2007; Yogendra et al. 2014).

It should be noted that herbivore preference is not exclusively determined by polar secondary metabolites, as detected by LC-qToF-MS. For example, *S. dulcamara* is known to produce and induce other defences upon herbivore feeding, such as polyphenoloxidases, peroxidases and protease inhibitors, which have not been measured here. These compounds mainly affect the plant’s nutritional value, but may also affect the spatial distribution of herbivores through the season (Nguyen et al. 2016; Viswanathan et al. 2008). Additionally, *S. dulcamara* successfully deploys indirect defences against slugs. Herbivore damaged plants produce extrafloral nectar from the wounds which attracts ants (Lortzing et al. 2016). In our common garden experiment, only a few ants or other predators were observed, probably because ant nests were removed when preparing the area. This makes it unlikely that differences in herbivore abundance or damage in this experiment are correlated with differences in nectar secretion and ant attendance. However, this does not rule out that variation in other defence traits, such as leaf toughness or trichome density (Agrawal and Fishbein 2006), may have contributed to the observed patterns.

The intraspecific variation in GAs that was reported for *S. dulcamara* before (Eich 2008; Mathé 1970) now also includes UACs as related compounds (Calf et al. 2018). The fact that three plants of the UAC-rich accession ZD11 did not survive the season due to excessive gastropod damage illustrates the potential consequences of relatively small chemical differences. It is expected that natural chemical variation among plant populations reflects adaptation to the locally dominant herbivores, in terms of inflicted damage (Kalske et al. 2012; Laine 2009; Scriber 2002). For gastropods and insect herbivores, differences in the herbivore community composition may concur with differences in local abiotic conditions among *S. dulcamara* populations (Zhang et al. 2016). Slugs and snails generally require moist conditions and intermediate temperatures (Astor et al. 2017). In dry natural populations, such as the sand dunes where ZD11 originates from, gastropods may be less abundant than specialist insects (Calf and van Dam 2012). The low abundance of gastropods in these populations may provide a ‘window of opportunity’ for chemotypes producing UACs to survive and propagate. This hypothesis is supported by the fact that only two accessions containing UACs were found, both from the same coastal dune population (OW Calf, pers. obs.). Closer monitoring of natural feeding damage incurred by UAC-rich accessions in different natural populations would be needed to test the role of abiotic conditions in survival of this chemotype. The genetic mechanisms underlying the GA polymorphism in *S. dulcamara* can be elucidated using our knowledge of their chemistry and biosynthesis in tomato and potato. *S. dulcamara* is closely related to these crop species and the genetic mechanisms for GA biosynthesis seem to be highly conserved (Cardenas et al. 2015; Eich 2008; Itkin et al. 2013). Further studies comparing genomes or transcriptomes of GA and UAC chemotypes will allow us to dissect the GA-biosynthetic genes contribute to this chemical polymorphism.

One of the first students of chemical ecology, Ernst Stahl, observed already 130 years ago that plants produce chemical defences to protect themselves against slug and snail feeding (Stahl 1888, see also the historic review by Hartmann 2008). Our study is in line with this and other studies, which show that gastropods may constitute a severe selection pressure in the field, especially by ‘weeding out’ specific seedlings (Rathcke 1985; Strauss et al. 2009). We also show that generalist gastropods and specialist insects may impose different selection pressures on chemical diversity. Heterogeneity in herbivore communities often concurs with abiotic differences among the habitats that plant species with broad ecological amplitudes can occupy. Together with random molecular events, such as gene mutation or duplication, this may be the reason for the intraspecific chemical diversity we observe in *S. dulcamara* and other plant species today (Hartmann 1996).

## Electronic Supplementary Material

ESM 1 (XLSX 367 kb)
